# High Throughput Tools to Access Images from Clinical Archives for Research

**DOI:** 10.1007/s10278-014-9733-9

**Published:** 2014-10-15

**Authors:** Shawn N. Murphy, Christopher Herrick, Yanbing Wang, Taowei David Wang, Darren Sack, Katherine P. Andriole, Jesse Wei, Nathaniel Reynolds, Wendy Plesniak, Bruce R. Rosen, Steven Pieper, Randy L. Gollub

**Affiliations:** 1 Research IS and Computing, Partners HealthCare, Charlestown, MA 02129 USA; 2 Laboratory of Computer Science, Massachusetts General Hospital (MGH), Boston, MA USA; 3 Department of Radiology, Massachusetts General Hospital (MGH), Boston, MA USA; 4 Department of Radiology, Brigham and Women’s Hospital (BWH), Boston, MA USA; 5 Beth Israel Deaconess Medical Center (BIDMC), Boston, MA USA; 6 Department of Psychiatry, Massachusetts General Hospital (MGH), Boston, MA USA; 7 Isomics, Cambridge, MA United kingdom; 8 Department of Meridian and Acupuncture, Collaborating Center for Traditional Medicine, East-West Medical Research Institute and School of Korean Medicine, Kyung Hee University, 26 Kyungheedae-ro, Dongdaemun-gu, Seoul, 130-701 Republic of Korea; 9 Information Systems, Partners HealthCare, Charlestown, MA USA

**Keywords:** Medical images, Informatics, Secondary research use of medical records

## Abstract

Historically, medical images collected in the course of clinical care have been difficult to access for secondary research studies. While there is a tremendous potential value in the large volume of studies contained in clinical image archives, Picture Archiving and Communication Systems (PACS) are designed to optimize clinical operations and workflow. Search capabilities in PACS are basic, limiting their use for population studies, and duplication of archives for research is costly. To address this need, we augment the Informatics for Integrating Biology and the Bedside (i2b2) open source software, providing investigators with the tools necessary to query and integrate medical record and clinical research data. Over 100 healthcare institutions have installed this suite of software tools that allows investigators to search medical record metadata including images for specific types of patients. In this report, we describe a new Medical Imaging Informatics Bench to Bedside (mi2b2) module (www.mi2b2.org), available now as an open source addition to the i2b2 software platform that allows medical imaging examinations collected during routine clinical care to be made available to translational investigators directly from their institution’s clinical PACS for research and educational use in compliance with the Health Insurance Portability and Accountability Act (HIPAA) Omnibus Rule. Access governance within the mi2b2 module is customizable per institution and PACS minimizing impact on clinical systems. Currently in active use at our institutions, this new technology has already been used to facilitate access to thousands of clinical MRI brain studies representing specific patient phenotypes for use in research.

## Background

The increasing accessibility of electronic healthcare data is transforming the ability of biomedical scientists to use information collected during the course of routine clinical care to advance knowledge about health and disease as well as to facilitate improvements in healthcare delivery. Paradoxically, the field of medical imaging, which has led the medical community in transition to digital information acquisition and storage, has lagged behind in efforts to instrument electronic healthcare records for secondary use by the scientific community in research discovery and clinical improvements [1]. Thus, there is an opportunity that has yet to be realized for using imaging studies in clinical research, imaging studies being potentially one of the most complete renditions of the human phenotype. Quantitative, noninvasive characterization of the human phenotype in healthy and disease states is now possible through the use of biomedical imaging in ways that were previously unachievable. Often, imaging modalities are used to assess trauma and acute internal conditions that are inaccessible to or would be impractical for other forms of phenotypic interrogation such as surgical exploration or laboratory testing. Such conditions are often difficult or impossible to investigate with animal models and would be unethical to replicate in volunteers. Large Picture Archiving and Communication Systems (PACSs) have millions of studies that could provide considerable insight into such conditions. However, this wealth of richly detailed phenotypic data currently remains difficult to obtain for investigators with a variety of domain specialties seeking to mine clinical data for fundamental understanding of disease or to generate tools that improve the diagnostic power of medical images. Traditionally, only the staff closely associated with the clinical data collection process had access to the data and the domain knowledge needed to correctly interpret it. To the best of our knowledge, this is the first report of an open source software solution that enables an institution to instrument their radiology archives so that appropriately credentialed investigators can access medical images for research.

A clinical PACS is designed to act in the service of routine clinical care and not for the general requirements of research. A typical PACS is optimized for radiology reading room workflow that is study-centric, and for enterprise access that is patient-centric, but not for searching for patients and studies with a particular disease. Thus, it is difficult to search for classes of human phenotypes associated with the images. Rather, this information, when available, is in coded from within the various electronic medical record systems in use at an institution. There are several important efforts underway to provide language that can be used for the classification of medical images, such as RadLex [2–4] and a number of software platforms that allow manual annotation of studies [5, 6] for future searching by metadata. However, these features are not widely available and there has been slow adoption by commercial systems.

In addition to the lack of phenotypic indexing in PACS, the images are not typically made available for research studies because of concerns around impact on clinical performance and security issues. Systems are not devised to allow individual investigators to gain access to only specific patients for a given study even when access has been granted by their Institutional Review Boards (IRBs). Health Insurance Portability and Accountability Act (HIPAA) allows the IRB to grant a waiver of consent for specific sets of patients for approved studies, but this is difficult to enforce in a PACS environment and cannot be audited by existing software. One way around these difficulties is to put an analyst in charge of screening and obtaining the studies for an interested party, an approach that does not scale well for hundreds of researchers requesting thousands of studies.

These are serious limitations, because many current and future clinical research studies rely on medical images for quantitative metrics for assessing diagnosis, making prognostic assessments, and for tracking treatment response and/or outcome. Images are used to quantify disease burden such as tumor volume, inflammation, hemorrhage, and infarction in tissues [7–12]. Serial imaging is used to quantify the outcome of interventions, such as changes in tumor size in response to chemotherapy in cancer patients [13] or changes in cerebral perfusion following thromboembolic therapy in stroke patients [14–18]. Importantly, medical images are providing an increasing number of sensitive diagnostic approaches to disease, such as the use of susceptibility weighted and diffusion tensor magnetic resonance imaging (MRI) in the evaluation of brain trauma [19]. Quality of care determinations, such as follow-up for tuberculosis therapy and assessment of catheter placement are based upon imaging studies in many institutions [20, 21]. But perhaps the most compelling research is in the natural history of disease progression, and the ability to look at early imaging in diseases that manifest later in life.

To address the logistical and regulatory challenges of identification and access to valuable medical image data for clinical translational research, we have developed the Medical Imaging Informatics Bench to Bedside (mi2b2) open source software. Mi2b2 software is an addition to the Informatics for Integrating Biology and the Bedside (i2b2)-based bioinformatics infrastructure (www.i2b2.org) [22]. As one of the sponsored initiatives of the NIH Roadmap National Centers for Biomedical Computing (http://www.bisti.nih.gov/ncbc/), i2b2 is the software platform adopted and extended by the Clinical Translational Science Center at Harvard (the Harvard Catalyst) to enable patients and patient materials to be discoverable and securely available for clinical translational research. A necessary and significant prerequisite is that the electronic medical record data from the hospital transaction systems be extracted and loaded into a database that is designed to perform the analysis. I2b2 is a site independent successor to the Research Patient Data Registry [23], which was custom-built for use at Massachusetts General Hospital (MGH) and Brigham and Women’s Hospital (BWH). Once the i2b2 servers are in place, the i2b2 workbench provides the way to perform systematic electronic health record analyses in order to assess phenotypes of patients and obtain sensitivity and specificity measurements of the extracted patient characteristics. Each characteristic is formulated as a definition that is validated with medical record review [24, 25]. These characteristics may be determined by coded diagnoses, medications, laboratory values, or health outcomes as well as annotations from the unstructured data through natural language processing (NLP) or human review [26]. NLP can be used to extract lifestyle and environmental factors, such as nutrition status, family history, and a broad host of other variables of interest. Data from NLP can also be used to validate coded information, as well as radiology and EKG findings and other diagnostic test results in the patient record. The analysis can be automated for different phenotypes in order to be performed recurrently once the definitions are encoded [27].

The mi2b2 software described in this report allows researchers to locate and retrieve medical images from PACS for subjects that have been identified via i2b2 or any other software platforms. Access to this data is provided in a manner that is compatible with the technical infrastructure and regulatory policies in place at our institutions. Because our collaborating institutions include departments with PACS from the vendors with a large market share (e.g., General Electric, AGFA, and Fuji), our solutions for image retrieval are immediately applicable to the majority of institutions. Our approach was to create a standard-based server (DICOM and web services) that can be readily deployed and integrated into the clinical imaging workflow of any modern radiology department and a rich Java client that can be configured to be a request agent for each project. As major research centers and tertiary care facilities, the administrative and regulatory environment at our institutions represents what we consider to be a widely accepted standard for patient protection and secondary use of clinical data.

## Methods

### Software Design Overview

The mi2b2 software meets a number of design goals that address the needs of members of the clinical translational research community (hereafter referred to as “users”). First, mi2b2 is a tool that empowers researchers to search, identify, review, and retrieve DICOM images from their institutional PACS. The software can be configured to support a single PACS within one institution or multiple PACSs within multiple affiliated institutions that are governed under a single IRB. Second, the mi2b2 software allows users to transfer selected images to a secure disk storage resource. Third, user access to the PACS is managed to minimize impact to clinical activities of the PACS. Importantly, the mi2b2 software and implementation policies ensure that user access complies with IRB regulation, is compliant with HIPAA and Office for Human Research Protections (OHRP) rules, and supports auditing. In addition, the mi2b2 software has robust informatics support to enable sophisticated data mining to identify medical images of interest. Finally, the mi2b2 software is based on industry standards for computing and medical informatics, is easy to deploy, and is shared with the scientific community under an open source license.

**Fig. 1:**
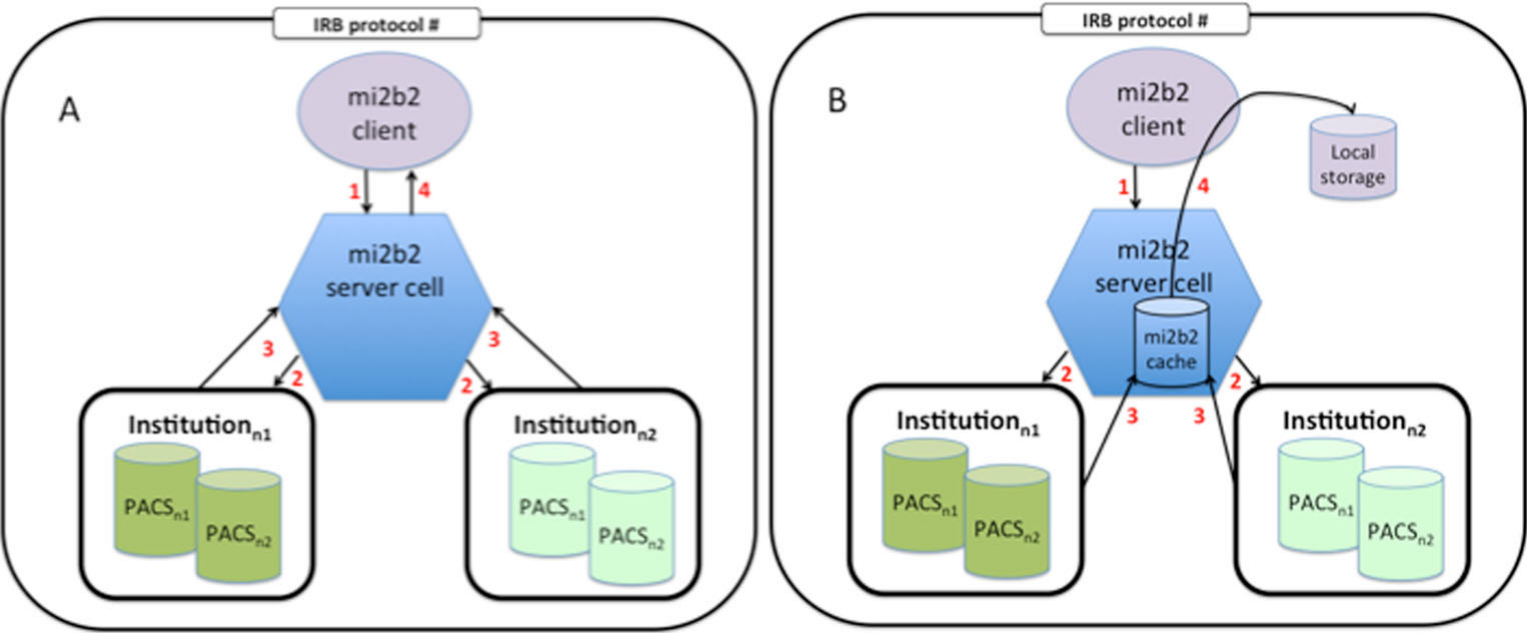
This schematic diagram shows how the mi2b2 software communicates with multiple institutional PACSs. In part *A*, we show the user initiating a query from the mi2b2 client for patient or study information (*1*). The mi2b2 server cell translates this query into a C-FIND request across the appropriate institution(s) and PACSs (*2*). The PACSs return information to the mi2b2 server cell (*3*) and the server cell formats and returns this information back to the mi2b2 client (*4*) for viewing. In part *B*, we show the user initiating an image request from the mi2b2 client (*1*). The mi2b2 server cell queues that request and then issues C-MOVE commands to the appropriate PACSs in accordance with the governance settings (*2*). The PACSs move the image data into a secure, central cache within the mi2b2 server cell (*3*). The user is notified that the images are available in the cache and he/she initiates the transfer of the image data through the mi2b2 client to the user specified local storage (*4*). Note that all steps must be specifically covered by an Institutional Review Board approved protocol

The mi2b2 software uses a client-server architecture where functionalities to find and transfer images are provided via web services (Fig. 1). The server acts as an intermediary between clients and the image data in the institutional PACS. This allows the mi2b2 server to moderate user access to the PACS, log data for HIPAA and OHRP compliant audits, restrict access to only IRB-approved records, and maintain managerial and administrative control. The audit trails and security protocols, including the ability to transfer encrypted images, are in compliance with HIPAA and research privacy protocols. The client serves as an interface by which users can issue commands to identify and transfer images from PACS to another DICOM entities or to their own secure disk space. The server provides a temporary storage cache that holds all transferred images retrieved from the PACS as a result of user requests. User projects are granted their own cache and individual users can interrogate the images within their own cache to decide whether to download them to their own secure disk space, to move them to other DICOM entities, or to delete them from their cache. The mi2b2 server’s configuration allows administrators to define how much cache space is allocated to each project, where several users under the same IRB may share space. When the allotted cache is full, studies in their cache need to be deleted by the user to create free space before additional studies can be accessed.

### IRB and Auditing Support

Central to the vision of the mi2b2 software is a robust way for administrators and healthcare entities to control and audit user activity that accesses both images and related protected health information on clinical archives. We do not attempt to de-identify or anonymize data sets [28] but instead leave this responsibility to the IRB-approved project members. The mi2b2 software ensures the privacy of all patients by limiting access to the data to only trusted users who are named on the associated IRB proposal. Per institutional policy, it is required that all named users will have up-to-date Collaborative Institutional Training Initiative (www.citiprogram.org) certification or equivalent and commit to following all behavioral and technological guidelines of the institution for protecting the data (e.g., password protected, encrypted computers, hard disks within institutional firewalls, etc.). Both the i2b2 and mi2b2 software tools are developed with these security measures as well as ex post review methods, including audit trail mechanisms for all image files accessed.

The mi2b2 software accomplishes this in two ways. First, mi2b2 limits user queries and requests for images to a predefined list of medical record numbers. This list is tied to an IRB protocol approval and may contain an expiration date to automatically stop PACS access when the approval has ended. Second, every query a user performs for patient information and study information is recorded in the database. The date and time of image retrievals from the mi2b2 cache is also tracked. At any time, administrators may audit mi2b2 activity by querying the appropriate tables in the database.

### Server

The software that communicates with the PACS resides on a dedicated server, configured by and only directly accessible by mi2b2 administrators. The server can either be deployed on dedicated hardware or configured as a virtual machine. The mi2b2 server creates a queue for each project, which includes the set of users under the same IRB, and sends study C-MOVE retrieval requests to the PACS in a round-robin fashion in order to serve all projects fairly. A retrieval request corresponds to one radiological study. If a maximum number of retrievals are active, no new requests can be sent. This limit prevents the mi2b2 server from monopolizing the PACS communication as some studies can take a long time to transfer (e.g., those physically stored in off-line archives). The limit additionally prevents the mi2b2 server from flooding a PACS with requests creating a bottleneck situation or downtime. The transfer rate between PACS and the mi2b2 server can be set to allow a specific number of studies per minute (or hour) and a specific number of concurrent downloads. The scheduling module is sufficiently flexible to allow different governing rates at times that might be associated with high and low PACS utilization (e.g., slower or no access during peak clinical activity and highest access during nights and weekends). These governing parameters for mi2b2 server’s access behavior to PACS are typically negotiated directly with the responsible parties within the radiology departments and are coded in a mi2b2 configuration file. Parameters enforcing IRB restrictions on accessible patients for each project are also contained in the configuration file.

### Client

**Fig. 2:**
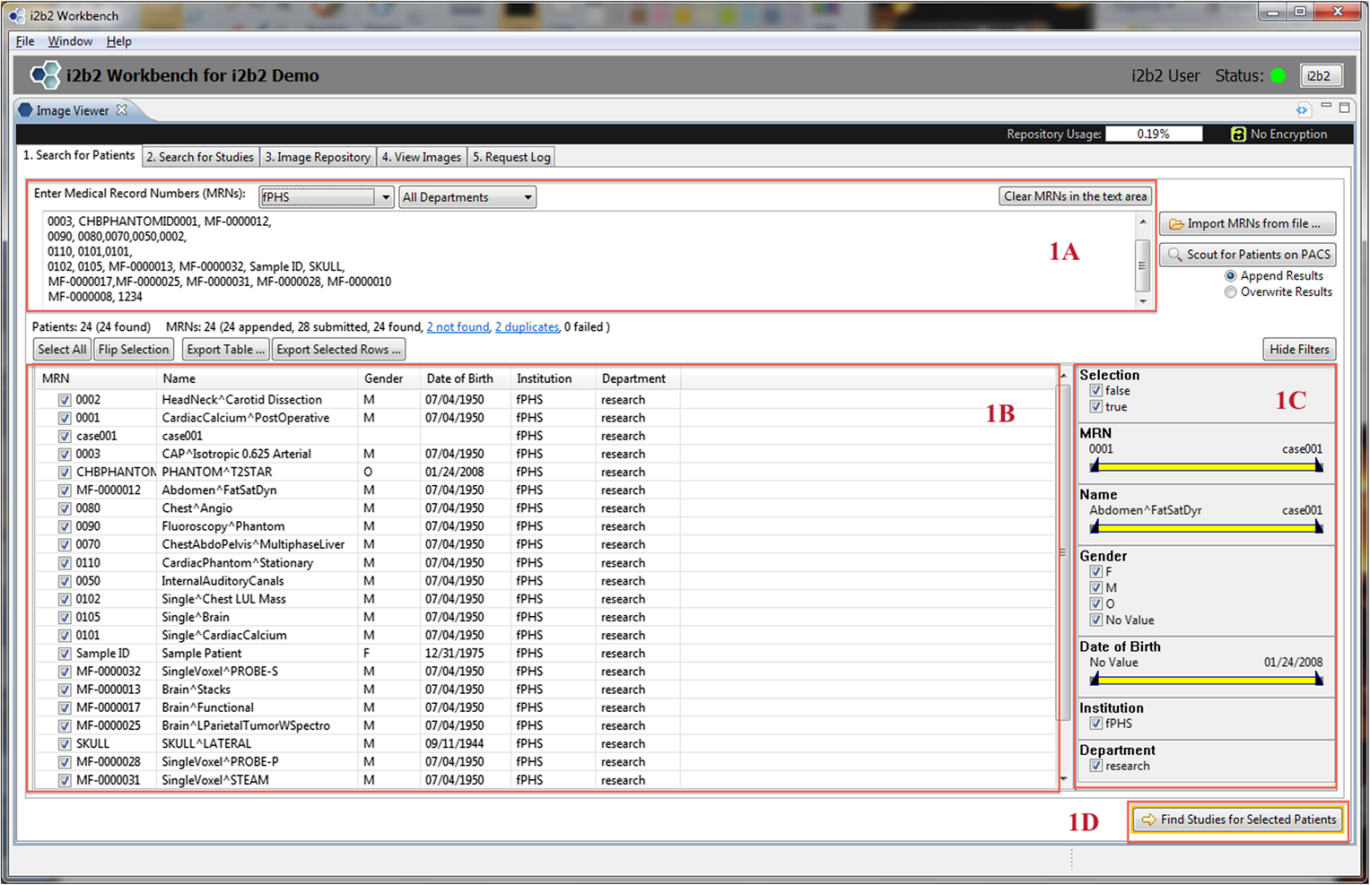
This is a screen shot of Tab 1 (*Search for Patients*). It shows a list of MRNs (*1a*) that are searched against the connected PACS. Search results (*patients*) are shown in the table below (*1b*). Controls on right allow users to further filter results (*1c*). Users initiate finding studies by clicking on “Find Studies for Selected Patients” button on the bottom right (*1d*). The formats of the MRNs vary because these are “TEST” MRNs, not truly linked to real patients

**Fig. 3:**
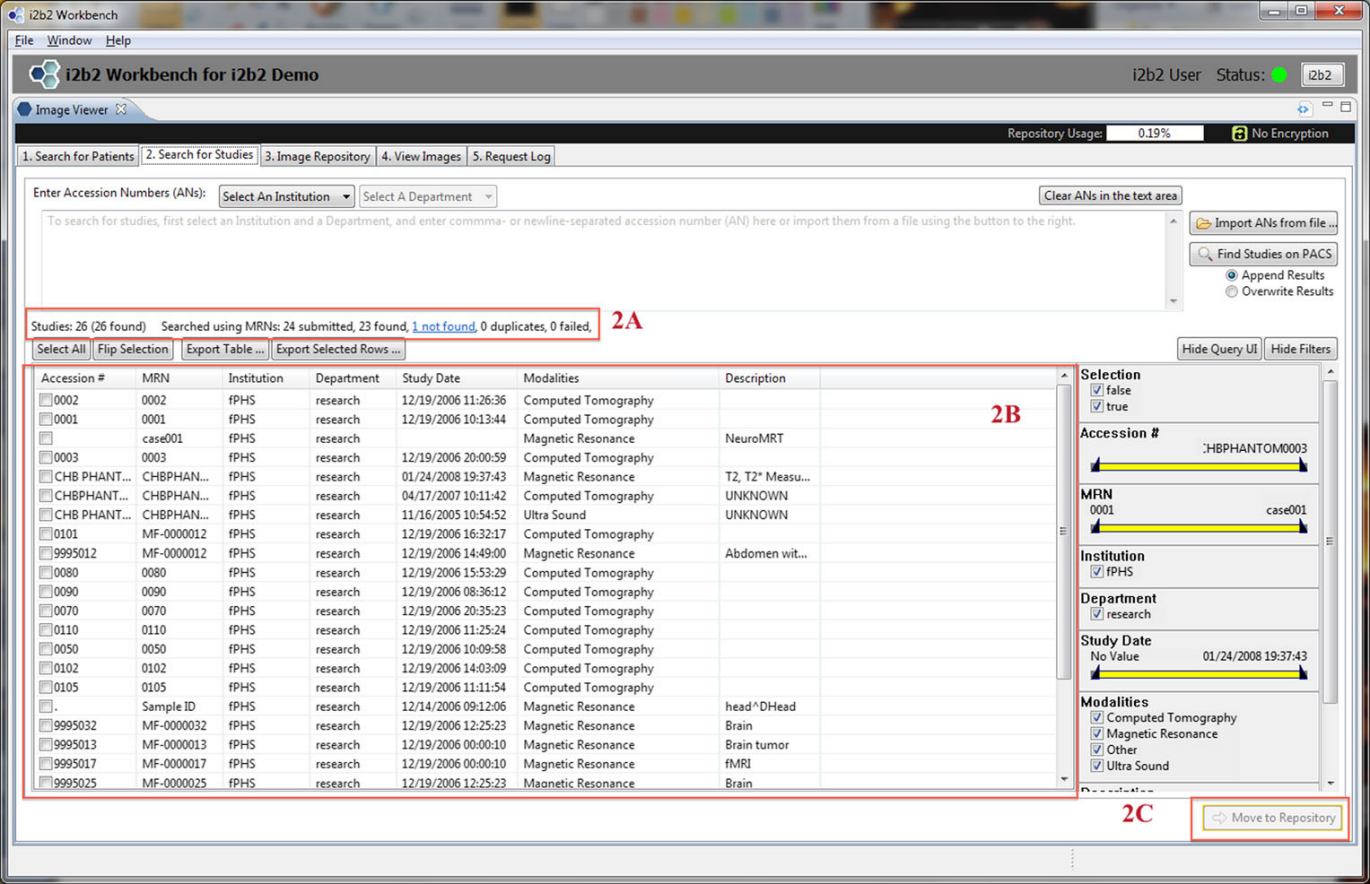
This is a screen shot of Tab 2 (*Search for Studies*). It illustrates how the patients previously selected in Tab 1 are used to search for studies (*2a*). Resultant studies are shown in the main panel (*2b*). Users can select desired studies and press “Move to Repository” to initiate a C-MOVE to move studies from the PACS to mi2b2’s cache (*2c*). The formats of the accession numbers vary because these are “TEST” accession numbers, not truly linked to real patients

**Fig. 4:**
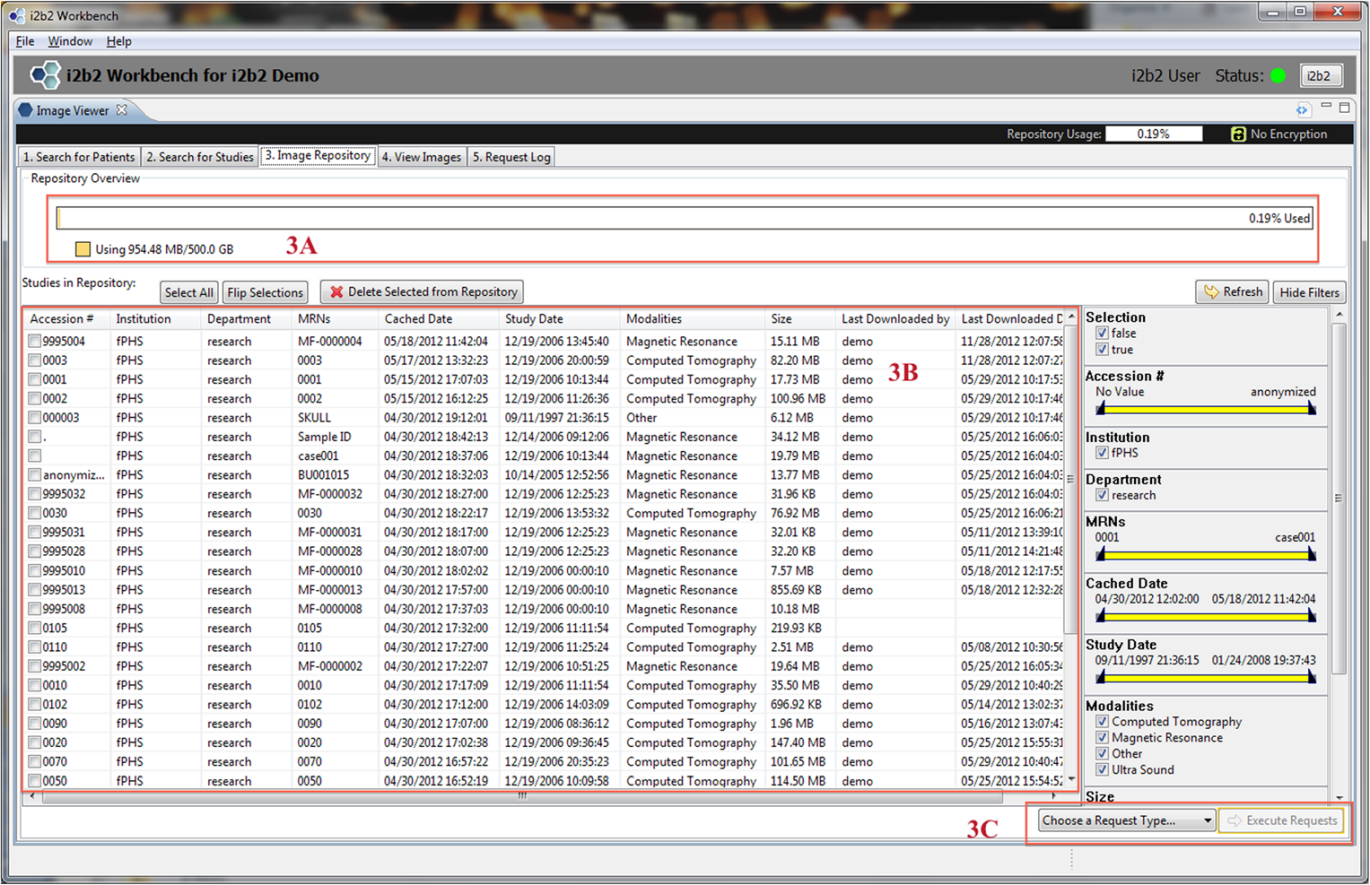
This is a screen shot of Tab 3 (*Image Repository*) showing all studies moved to the mi2b2 cache (see Fig. 3). The used and available capacity of the cache is displayed on top (*3a*), and the list of available studies is shown in the main panel (*3b*). Users can manage their cache usage by deleting studies. Selected studies can then be copied to other connected services such as XNAT, OsiriX, or to a local machine by selecting from the drop down box “Choose a Request Type…” on the bottom right (*3c*)

**Fig. 5:**
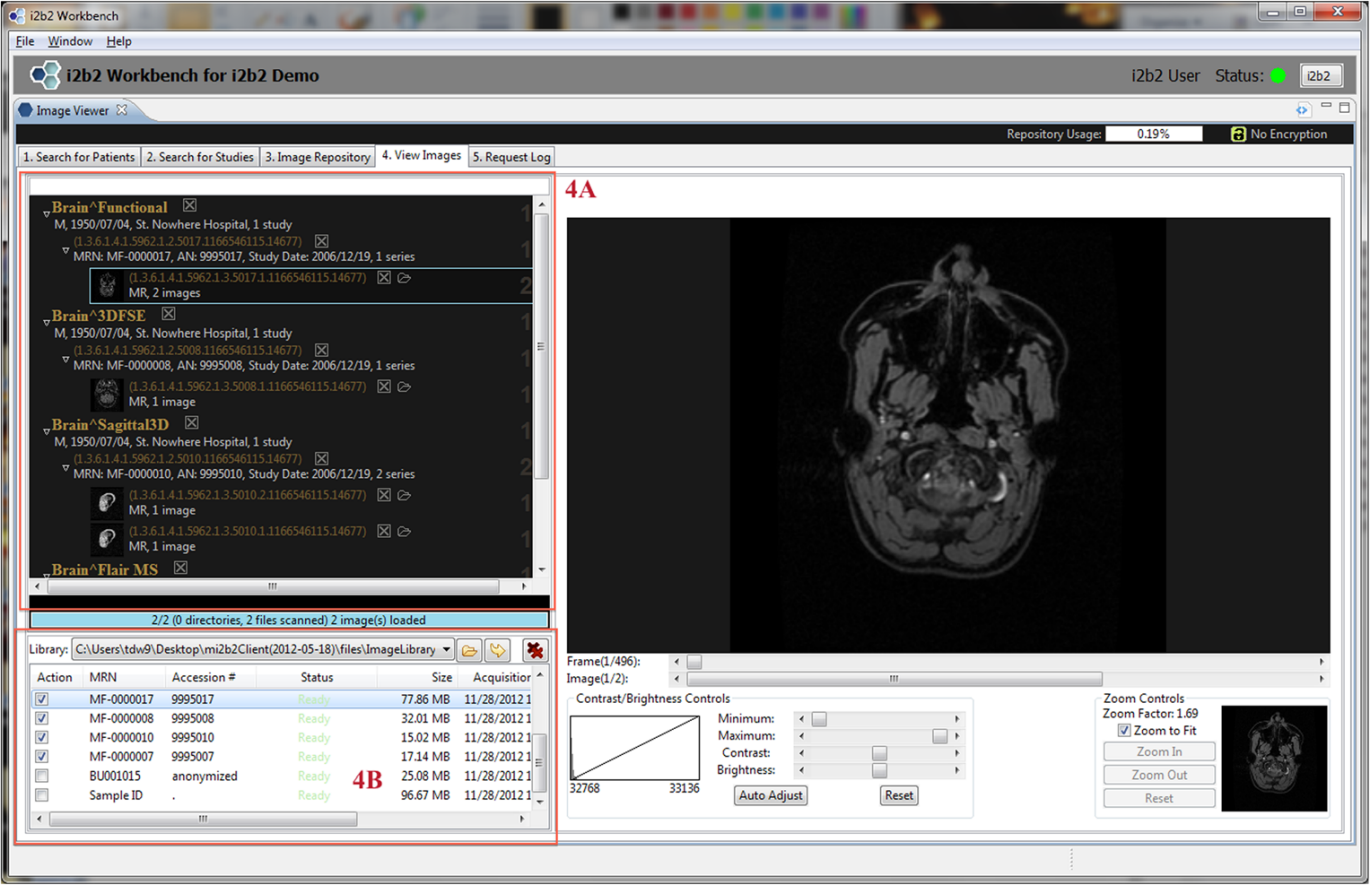
The simple DICOM image viewer in Tab 4 of the client lets users view downloaded studies. Selected studies are organized on top left by patient ID, study UID, series UID, and image ID (*4a*). The bottom left panel shows a list of studies already downloaded to local machine (*4b*)

**Fig. 6:**
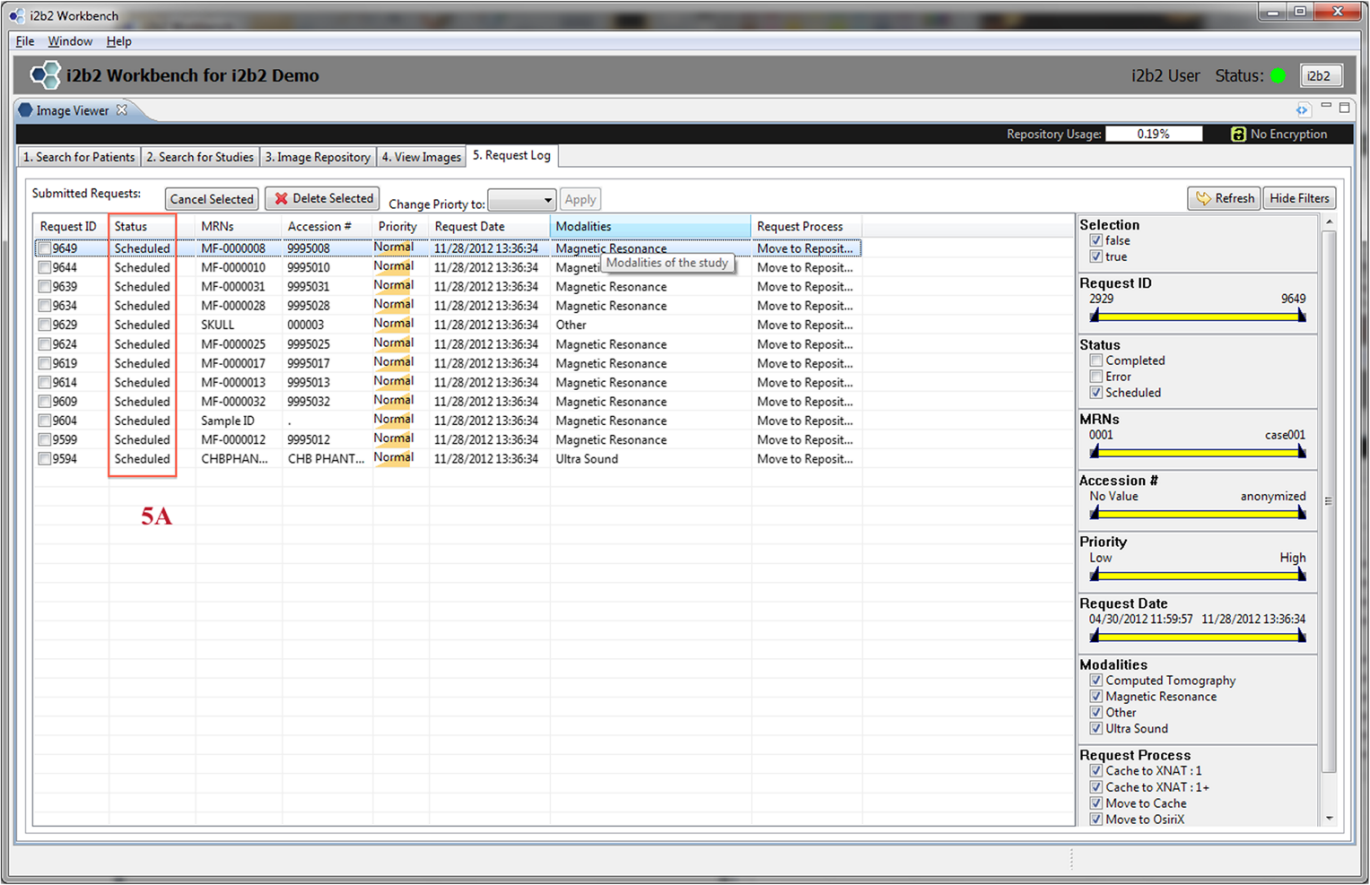
Tab 5: All requests, such as a C-MOVE, made to mi2b2 are placed in the Request Log in Tab 5. Users can track whether a request is scheduled, processing, completed, or has failed (*5a*)

The mi2b2 client supports four main functionalities: (1) selecting imaging studies, (2) submitting and managing requests to transfer these studies (from PACS to the cache on the mi2b2 server), (3) downloading and viewing images, and (4) managing individual server cache. The interface is comprised of five tabs to guide users through querying for studies using multiple criteria, managing query results, requesting clinical data and monitoring retrieval status, previewing retrieved data, and transferring data to other DICOM entities (e.g., other PACSs, Extensible Neuroimaging Archive Toolkit (XNAT) [29], OsiriX (http://www.osirix-viewer.com/), or to local disk space). Specifically, Tab 1 (Search for Patients) and Tab 2 (Search for Studies) shown in Figs. 2 and 3 allow users to find radiology studies by medical record number (MRN) and accession number (AN) respectively. This functionality is supported by a C-FIND request issued from the mi2b2 server. Tab 3 (Image Repository) lets users manage their partition of the server cache (Fig. 4). Tab 4 (View Images) lets users manage downloading images from the mi2b2 cache to their local machine and to view downloaded images using a simple DICOM image viewer (Fig. 5). Finally, Tab 5 (Request Log) allows users to view and manage image transfer requests (Fig. 6). The mi2b2 client user interface is designed to permit flexible navigation among workflow steps and to be compatible with existing i2b2 user interface paradigms.

A typical workflow starts with a successfully logging in to the mi2b2 client. Users first select which PACS to retrieve from by selecting an institution (e.g., a hospital) and, optionally, a department (e.g., radiology). All institutions and departments are preconfigured when the mi2b2 server is set up. If users have a list of MRNs, the list can be readily entered in Tab 1 (Search for Patients) to obtain only patients that have radiology studies (Fig. 2). Users then submit a query to the PACS for specific patients from that list. A list of radiology studies associated with the queried patients is subsequently displayed in Tab 2 (Search for Studies) automatically. These studies are identified by their accession numbers. If users have a list of ANs for specific imaging data they are interested in, they can skip Tab 1 (Search for Patients) and start with Tab 2 (Search for Studies). Finally, users then select studies they are interested in to initiate a request to retrieve them from the PACS to the mi2b2 cache.

Newly submitted requests are found in Tab 5 (Request Log), where users can track request status (Fig. 6). A successfully completed request adds new entries in Tab 3 (Image Repository), where a view of transferred studies in the user’s cache is presented (Fig. 4). In Tab 3 (Image Repository), users can see how much space they are using in their cache and, if necessary, delete studies from cache to make room for new requests. Users can also download any study to their local disks or transfer them to other DICOM entities. Downloading to a local disk can be monitored in Tab 4 (View Images), where progress for each download is displayed (Fig. 5). In addition, any locally stored study, including those downloaded with encryption, can be opened for viewing in Tab 4 (View Images).

The mi2b2 client supports faceted search using filters. Most tables in the mi2b2 client have a filter control that allows the user to limit the display of results. Each column in the table can be filtered by a range, and multiple columns can be filtered at the same time to find, for instance, studies dated within a certain time interval, of a specific modality, and/or from a particular institution or department. The filters are set up as either a range slider or a set of checkboxes. These widgets afford users the ability to perform conjunction-or-disjunction queries over these facets (e.g., “Find studies that are performed from 01/01/2009 to 01/01/2012 AND are from Hospital X or Hospital Y AND are X-ray or ultra sound or magnetic resonance images.”) These filters automatically show up in Tab 1 (Search for Patients) and Tab 2 (Search for Studies) when MRN or AN searches are performed (Figs. 2 and 3). They are also available in Tab 3 (Image Repository) and Tab 5 (Request Log) when users click on the “Show Filters” button (Figs. 4 and 6).

An important security feature of the mi2b2 client is the ability to allow users to encrypt their image data when downloading to their local machines. This is a critical feature since the overall design of the project requires each user to be responsible for maintaining the security and privacy of the accessed data, without de-identification of the images by mi2b2. The mi2b2 client uses 128-bit AES encryption [30] based on a user-defined key. The encryption prevents those without the key access to private, identifiable patient information contained in the images. When the users enable downloading with encryption, the download stream is encrypted as it is saved onto the users’ hard disks. This ensures that at no point will there be a copy of an unencrypted image on the target disk. The file names of the study, series images are additionally obfuscated on the file system. The image file names are renamed while preserving the order of the images. When an image is decrypted, it is also done in a way that no unencrypted portions of the image are saved on the hard disk. The only unencrypted images are on screen and in the machine’s volatile random-access memory.

The mi2b2 platform is both easy to deploy and easy to customize. Once the mi2b2 server is configured and started, users can download the workbench from the mi2b2 website (www.mi2b2.org) and be up and running within minutes. Mi2b2 is built using open source tools and standards that are widely supported by both the imaging and informatics communities. Mi2b2 is developed using the Java programming language and the Eclipse platform, with the server installation able to run on Microsoft Windows and Linux OS and the client able to run on Microsoft Windows and Macintosh platforms. Mi2b2 incorporates code from the dcm4che2 toolkit (http://sourceforge.net/projects/dcm4che/), an open source Java-based suite of code to work with DICOM networking and data objects. Advanced image viewing and manipulation is supported by the use of ImageJ (http://rsbweb.nih.gov/ij/) and the Java Advanced Imaging API; both of these libraries are open source and freely available. Mi2b2 itself is freely available to the community under the i2b2 open source Software License (https://www.i2b2.org/software/index.html). This license allows institutions to use and customize the software to fit the unique needs of each site.

## Results

Mi2b2 has been deployed for early adopter researchers with IRBs in place in three tertiary care institutions with diverse radiology IT infrastructures: MGH, BWH, and Children’s Hospital Boston. The institutions span three different PACS vendors: AGFA IMPAX (www.agfahealthcare.com), General Electric (www.gehealthcare.com/usen/img_info_systems), and Fuji Synapse (www.fujifilmusa.com/products/medical/radiology). Minor differences between sites prompted refinements to the project requirements, such as configured limits on numbers of studies that were returned per patient request, and support for multiple different formats of the MRN. These differences have been addressed and mi2b2 software is successfully serving all three sites.

As of the writing of February 2014, mi2b2 has been used as a production system for 6 months by 24 users on 13 separate projects. There have been over 27,900 individual requests for studies from the PACS with approximately 26,000 of those requests completing successfully. With the exception of one night in late 2013 where an unforeseen bug in the software caused 1200 requests to fail, the overall success rate of the software has been steady at approximately 97 %. During this time, we have continued to modify the rules governing mi2b2’s access to the PACS; we have gradually increased the nightly download rates and the number of downloads that can be processed simultaneously. On average, mi2b2 downloads 198 studies per night; this number has continued to increase over time and is currently less than the maximum number of downloads allowed. It indicates that, on the majority of nights, mi2b2 processes the entire request queue. We have only recently started tracking the size of the individual requests that get downloaded from the PACS, but based on data from the first 6000 requests, the average download size per study is 97.3 MB with the largest study in that group at being just over 780 MB. Based on those numbers, it is estimated that mi2b2 has downloaded and processed over 2.4 TB of data to date.

## Discussion

In this article, we describe the features and use of the mi2b2 software, an integrated and standalone module of the i2b2 software suite of tools that facilitates repurposing all types of medical image data for secondary research and educational uses. This software addresses a critical need of researchers, busy radiology IT staff who are tasked with providing image data from cohorts of patients for clinical investigators, and medical image analysis software developers who require access to exemplar data sets for testing and validating new algorithms. By adhering to the DICOM standards for query and retrieval of images, the mi2b2 software can be broadly applied to all modalities and clinical applications.

By their nature, clinical PACS include the most detailed imaging studies for diseases and conditions where imaging has been determined to have the greatest impact on patient care. The natural corollary is that detailed analysis of large collections of these images in the context of the complete medical record can be expected to yield significant insights.

As one example of the type of immediately valuable clinical image data that contains information that would be impossible or unethical to acquire prospectively in a controlled setting is the availability of “normal” MRI scans in infants and young children. It is clinically challenging to define “normal” in pediatric brain MRI scans because normal is a constantly moving target, changing rapidly with brain development [31, 32]. Age-related changes in MR contrast properties of the healthy brain are so dramatic that they compromise interpretation of pathological changes. For example, *gestalt* visual diagnosis of brain injury is difficult in the developing brain due to the rapidly changing appearance of normal, making it difficult for even experienced neuroradiologists to detect subtle abnormalities when relying on visual interpretation alone. Pediatric data sets are often difficult to acquire, especially on normative subjects, and multiple data sets on subjects spanning 0 to 6 years in age are needed to characterize normal brain development. We estimate a minimum of ten studies at monthly intervals totaling over 700 imaging studies would be required to build a collection of age-specific normative imaging studies. Prospective case collection would be costly. Whereas retrospective collection of these cases for inclusion in an age-representative atlas could be performed more cost effectively using the mi2b2 tool and analyst time. We are currently pursuing this opportunity with the goal of providing the resultant normative atlases to the clinical community [33].

The mi2b2 software provides many improvements to existing methods for accessing medical images outside of clinical practice. Most importantly, as configured, the mi2b2 software does not impact clinical operations. Although many research-oriented viewing workstations, visualization, and analysis software tools (e.g., OsiriX, ClearCanvas, http://www.clearcanvas.ca/dnn/, or 3D Slicer http://slicer.org) can connect to a PACS, clinical radiology departments need assurances that retrieving images for research will not interfere with clinical use. In a typical research project, many thousands of images may be obtained for hundreds of studies, and often in usage patterns that are quite different from conventional clinical workflow (research requests for images often delve deep into imaging archives, for example performing bulk requests for data that has been archived to off-line storage). By using site-specific configuration files, the mi2b2 software governs access to the PACS with day and time granularity.

The mi2b2 software rigorously protects patient privacy and complies with all regulatory requirements while making valuable clinical images readily accessible to investigators for approved research. There are legitimate concerns for patient privacy when repurposing medical data for secondary research use (see [22]), and we have chosen to develop our software solution within a rigorous regulatory framework that follows best practices. This includes proper protocol review by the IRB, investigator training, and good information management practices all of which are supported by a thoughtful software design (e.g., optional encryption at time of image data transfer from the secure mi2b2 server) and a thoroughly tested implementation. Our policy decision to place ownership of de-identify or anonymize the medical images on research users is in line with an emerging consensus within the clinical data sharing research community [34–36]. Critical to our design was the provision of easily accessible audit logs of all image data retrieved from the clinical PACS to comply with federal privacy and local IRB regulations.

The mi2b2 software is platform independent with respect to PACSs. Our institutions represent a microcosm in which equipment from different PACS vendors, different policies for online, near-line, and off-line storage, and different radiology IT infrastructures were all accommodated early in the design and implementation process. Our experience suggests that mi2b2 software should be a system that can be deployed with minimal changes at most institutions.

The mi2b2 software enables users to do the work of accessing the data with minimal burden on PACS administrators and limited support from a central clinical IT infrastructure team. One of the main goals of the mi2b2 project was to empower clinical investigators and/or medical image analysis tool developers who want access to medical image data to do so as independently as possible. This access model fosters better control, better auditing, and better scalability—freeing PACS administrators from the task of servicing and policing research requests. Once our mi2b2 servers were setup to connect to PACS, our users were able to use the interface (with online user’s manual https://community.i2b2.org/wiki/display/mi2b2/mi2b2+User+Documentation) to perform searches via MRNs or ANs and successfully retrieve studies without the support of a PACS administrator and only minimal support from our central clinical IT team. That said, it is absolutely necessary to have institutional investment in this endeavor, with support from the radiology department, the IRB, and centralized IT department and expertise to set up the mi2b2 software as well as to implement specific site requirements.

There are many problems inherent in the use of clinically acquired medical image data that the mi2b2 software cannot solve. Most importantly, this software does not address the issue of how to identify specific patient cohorts with images of interest. It is challenging to parse medical records information to determine cohorts and to create well-defined diagnosis groups and even more difficult to identify appropriately matched control cohorts [37]. That task requires the use of other clinical informatics tools such as the Research Patient Data Registry [23] or the parent i2b2 software. However, for users who already have identified patients or even specific scans and are able to start with a list of MRNs or ANs, the mi2b2 software is fully functional for retrieving studies of interest. Another limitation that is inherent to accessing images stored in PACS using mi2b2 is that it is not possible to query for a specific imaging scan type or specific sequences within a modality. This is especially problematic for MRI scans because of the wide range of imaging parameters used during scan acquisition. Similarly, it is also not possible to determine image data quality or any details of the acquisition parameters prior to downloading the images from the PACS, so that researchers that require very specific scan sequences may need to filter through many examinations before arriving at an adequate cohort of usable data. For some research needs previously acquired examinations may not be of acceptable quality (e.g., inadequate spatial resolution, limited field of view, inappropriate orientation), may be too highly variable (many protocols are highly technologist dependent), or have too high an incidence of technical artifacts (motion, scan spiking, other artifacts) that do not preclude meaningful clinical interpretation but severely limit utility for quantitative image analysis. Last, storage and medical data update policies are up to the individual PACS administrators. HL7 messaging may update data after time of access.

Despite these limitations, we believe that there will be sufficient quantity of clinical image data of sufficient quantity to be of value to the medical research community. For example, in recently published studies from a set of demonstration projects, the i2b2 infrastructure was used to identify a specific subpopulation of patients with confirmed treatment resistant Major Depressive Disorder who had a clinically acquired MRI scan of the brain. While mi2b2 software was not used to obtain the scans, existing radiology departmental infrastructure was used, the studies clearly demonstrate that the quality of the medical image data was comparable to that collected in routine research studies in our institutions [38, 39].

It is our hope that there will be many institutions that will find the mi2b2 software useful and that by embracing the open source software model, additional feature opportunities will be discovered and a growing community of users will contribute a steady stream of improvements. The imaging plug-in for mi2b2 is available now (www.mi2b2.org) as an open source addition to the i2b2 software platform under the i2b2 open source license and is supported by the mi2b2 developers. Future releases of mi2b2 as well as updates will be released to the scientific community through this site as they are completed.

## Conclusion

Access to medical image data is essential to the community of medical image analysis algorithm developers and clinical scientists seeking to enhance the extraction of meaningful information from medical images to speed the development of quantitative imaging biomarkers. Delivery of this crucial infrastructure facilitates the clinical translation of image analysis advances into clinical practice by overcoming technological, regulatory, and cultural impediments that historically have drastically slowed progress in this domain. Unlike any other approach to acquire medical images for research purposes, the integration and deployment of the mi2b2 software uniquely provides a continuously renewing resource of valuable medical images for the modest cost of administration of the software. Most academic medical institutions have substantial and enduring investments in the delivery of specialized medical care including advanced imaging. Because these institutions strongly support innovation in medical imaging technology, the mi2b2 project enables investigators to leverage this medical system cost as an investment into the advancement of quantitative biomarkers. As new mi2b2 based clinical translational research projects are established, they can continually replace older data with state of the art acquisitions having improved image quality and dimensionality and thus increase the accuracy and precision of quantitative results. We hope that by making the system freely available, we will have contributed to the research of others seeking to use the incredible opportunities of clinical image repositories to understand a wide range of human disease and treatment options.
